# Measurement of Calcium Activity in Oral Fluids by Ion Selective Electrode: Method Evaluation and Simplified Calculation of Ion Activity Products

**DOI:** 10.6028/jres.105.030

**Published:** 2000-04-01

**Authors:** C. M. Carey, G. L. Vogel

**Affiliations:** American Dental Association Health Foundation, Paffenbarger Research Center, and National Institute of Standards and Technology, Gaithersburg, MD 20899-8546

**Keywords:** calcium activity, calcium ion-selective electrode, mineral saturation, plaque fluid, saliva

## Abstract

The activity of calcium in plaque fluid is needed to calculate the saturation level of that fluid relative to the tooth mineral. One method to determine the calcium activity in very small plaque fluid samples is by micro ion-selective electrode (ISE). Two commercially available calcium ionophores, a neutral-carrier and a charged-carrier, were evaluated in micro ISEs and compared to a commercially available macro ISE using saliva as a model for plaque fluid. The neutral-carrier containing ISEs gave results consistent with those of the macro ISE. Calcium activity measurements made with micro ISEs that contained the neutral ion-carrier of whole plaque samples and plaque fluid samples obtained by centrifugation of whole plaque showed that the activities did not change due to centrifugation. Estimates of the saturation with respect to hydroxyapatite were made from these measurements. A simplified calculation method is presented to estimate the ion activity product (*IAP*) of the calcium-phosphate minerals. The method is based on the relative abundance of some of the possible calcium-binding species and a fixed ionic strength for plaque fluid. Calculations show that within a normal pH range for plaque fluid (5.0 to 7.5) the differences in the *IAP* calculations for hydroxyapatite using the simplified method are less than those estimated from propagation of uncertainty calculations.

## 1. Introduction

The concentration of calcium in the fluid phase surrounding teeth is one of the primary factors that determines whether a tooth remineralizes or demineralizes [1]. Specifically, it is the concentration of ionic calcium (the calcium not bound by anionic species, [Ca^2+^]) that is the relevant form of calcium in determining saturation within plaque fluid (PF) with respect to enamel minerals [2]. The introduction of the calcium ion-selective electrode (Ca-ISE) and the development of ultramicro ion-selective electrode methods for the measurement of calcium in nanoliter volumes of plaque fluid [2,3] makes it possible to directly determine the ionic calcium concentration in PF.

The measurement of [Ca^2+^] in PF by Ca-ISE has proven to be difficult due to several factors, which includes small sample size, protein poisoning, and interfering ions found in PF [4,5]. Although many of the difficulties inherent to Ca-ISE measurements have apparently been surmounted [2,6,7], a methodical evaluation of Ca-ISEs suitable for use in the analysis of PF has not been presented. In this report, two different commercially available calcium ionophores were used to fabricate micro Ca-ISEs, the responses of which were compared to that of a commercially available macro Ca-ISE designed for the analysis of blood and serum calcium. Whole pooled saliva was used as a model for plaque fluid. One potential advantage of ISE methods is the possibility of direct measurements of PF [Ca^2+^] without the need for sample centrifugation. Using micro Ca-ISEs that are shown to agree with the macro Ca-ISE in saliva determinations, comparisons of the pH and [Ca^2+^] between whole plaque and PF centrifuged from the same whole plaque samples are presented.

One important advantage that the Ca-ISE affords is that the activity of calcium ({Ca^2+^}) is directly determined. Note that in this report quantities in square brackets [ ] represent free ion concentrations and quantities in curly brackets { } represent ion activities, and thus s{Ca^2+^} = *γ*_Ca_^2+^ · [Ca^2+^], where *γ*_Ca_^2+^ is the activity coefficient. The quantity {Ca^2+^} can be used directly in the calculation of the ion activity product (*IAP*) with respect to any of the calcium-phosphate minerals. Therefore, *IAP* calculations made from the activities of the component ions, {Ca^2+^}, {H^+^}, {OH^−^}, $\left\{{\text{HPO}}_{4}^{2-}\right\}$ and $\left\{{\text{PO}}_{4}^{3-}\right\}$, can be used to evaluate conditions of saturation in PF with respect to enamel mineral. A simplified method for the calculation of *IAP*s with respect to calcium-phosphate minerals that uses the Ca-ISE determined value for {Ca^2+^} is presented. This calculation method is suitable for use in spreadsheet programs.

## 2. Materials and Methods

### 2.1 Sample Manipulation

Immediately after collection, the samples of saliva, whole plaque, and plaque fluid were placed in a mineral oil-filled dish equilibrated with a gas mixture containing a volume fraction of 5 % CO_2_ in nitrogen and saturated with water (5 % CO_2_−95 % N_2_−H_2_O_Sat_) to avoid evaporative loss of water or change in pH due to CO_2_ loss from the sample. Samples were stored less than 30 min before analysis. The physiological range for the partial pressure of CO_2_ (*p*CO_2_) in the parotid saliva has been reported to be between venous and arterial levels, with an average of approximately 5 % for a moderate salivary flow rate (0.25 mL/min) [8]. For this reason 5 % CO_2_−95 % N_2_−H_2_O_Sat_ gas mixture was used to equilibrate plaque fluid and saliva samples during measurements.

### 2.2 Collection of Saliva Samples

Fresh-pooled, unstimulated saliva samples were collected by expectoration from 15 individuals. The first 2 mL of saliva were discarded and the following 5 mL were analyzed. Saliva samples were analyzed for pH and {Ca^2+^} by ISE under 5 % CO_2_−95 % N_2_−H_2_O_Sat_ equilibrated mineral oil immediately after collection and without further sample preparation.

### 2.3 Collection of Whole Dental Plaque and Plaque Fluid Separation

Supragingival dental plaque was collected as described in [9] from the buccal surfaces of individual upper and lower molars. Plaque fluid was separated from the whole plaque by centrifugation at 117 680 m/s^2^ (12 000 *g*), 15 min, 4 °C and the fluid removed via micropipette [2]. All samples were placed under 5 % CO_2_−95 % N_2_−H_2_O_Sat_ mineral oil for analysis.

### 2.4 Electrode Construction

Reference, pH, and calcium selective microelectrode construction has been described previously [3]. Two types of calcium ion exchanger material were tested in the calcium microelectrodes: a neutral carrier, N,N′-di(11-ethoxycarbonyl)undecyl-N,N′-4,5-tetramethyl-3,6-dioxaoctane-diamide (ETH-1001) [10] available from World Precision Instruments^1^ (Cat# IE-200, WPI, Sarasota, FL) and a charged carrier, bis(di[4-(1,1,3,3-tetramethylbutyl)phenhyl]phosphate) (t-HDOPP, Cat# IE-202, WPI) [11]. The macro calcium selective electrode (ICA1, Radiometer, Copenhagen), that was used as the “standard electrode” had the same charged carrier material as above in its membrane. The sensing membrane was further protected from macromolecular interference with a dialysis membrane [12,13].

### 2.5 Ion-Selective Electrode Determination

#### 2.5.1 Calcium Activity Standards

Standards were made from a standardized CaCl_2_ solution (Fischer Scientific, Lanham, MD) with a 150 mmol/L KCl ionic strength background (similar to the ionic strength of plaque fluid [14]). The total calcium concentrations of the standards were (0.1, 0.5, 1.0, and 5.0) mmol/L for an activity of (0.033, 0.166, 0.331, and 1.625) mmol/L respectively. The activity of calcium in the standards was calculated from the ionic strength and using the Davies’ modification of the Extended Debye-Hückel Equation [15].

#### 2.5.2 Saliva

Ca-ISE analysis of saliva samples was carried out under 5 % CO_2_−95 % N_2_−H_2_O_Sat_ mineral oil. Samples were deposited on the surface of the inverted macro Ca-ISE. The micro Ca-ISEs and a micro reference electrode were positioned in the sample. In this manner the responses of two or more microelectrodes and the macro electrode could be measured simultaneously for paired analyses of the results. This method also eliminated the effect of reference electrode error because the measurements were simultaneous and had the same reference electrode.

#### 2.5.3 Whole Plaque and Plaque Fluid

Samples were placed in dishes filled with 5 % CO_2_−95 % N_2_−H_2_O_Sat_ mineral oil for microelectrode analysis. Calcium and pH microelectrodes and a micro reference electrode were positioned in the sample for simultaneous analysis [2,3].

### 2.6 Spectrophotometric Determination of Total Calcium and Total Phosphate

#### 2.6.1 Plaque Fluid

Nanoliter volumes (10 nL to 50 nL) of plaque fluid samples were analyzed as has been described [16] by use of precalibrated nanoliter pipettes for samples and standards. Total calcium ([Ca]_Tot_) determinations utilized the Arsenazo III reagent and a microspectrophotometer with a 2.3 μL cell volume [2,16,17]. The relative standard deviation (RSD) observed from this calcium method, determined by the repeated analysis of known samples, has been found to be less than 2 % [16]. The total phosphate ([PO_4_]_Tot_) concentration was colorimetrically determined by use of a phosphomolybdate method [17] and a microspectrophotometer [2]. The RSD of this phosphate analysis technique, determined by the repeated analysis of known samples, has been found to be less than 1 % [16]. Phosphate analyses for some PF samples were also made by capillary electrophoresis methods [18]. Paired analyses of the results from the colorimetric and capillary electrophoresis methods, to determine phosphate concentration in PF, indicated no significant difference between the values obtained by these techniques (significance level *a* > 0.1, *n* = 13 pairs, paired *t*-test).

### 2.7 Rigorous Calculation of the Ion Activity Product for the Calcium-Phosphate Salts

The ion activity products (*IAP*s) were calculated for the calcium-phosphate minerals with the measured quantities {H^+^}, {Ca^2+^}, [PO_4_]_Tot_, and the ionic strengths of the solutions. The calculation of the *IAP*s for the calcium-phosphate minerals required that the activities of H^+^, OH^−^, Ca^2+^, ${\text{HPO}}_{4}^{2-}$, and ${\text{PO}}_{4}^{3-}$, were known, as can be seen from Eqs. (1)–(5):

$$IAP_{\text{DCPD}}=\{{\text{Ca}}^{2+}\}\cdot \{{\text{HPO}}_{4}^{2-}\},$$ (1)

$$IAP_{\beta \text{TCP}}={\left\{{\text{Ca}}^{2+}\right\}}^{3}\cdot {\left\{{\text{PO}}_{4}^{3-}\right\}}^{2},$$ (2)

$$IAP_{\text{OCP}}={\left\{{\text{Ca}}^{2+}\right\}}^{4}\cdot \{H^{+}\}\cdot {\left\{{\text{PO}}_{4}^{3-}\right\}}^{3},$$ (3)

$$IAP_{\text{HAp}}={\left\{{\text{Ca}}^{2+}\right\}}^{5}\cdot {\left\{{\text{PO}}_{4}^{3-}\right\}}^{3}\cdot \{{\text{OH}}^{-}\},$$ (4)

$$IAP_{\text{ACP}}={\left\{{\text{Ca}}^{2+}\right\}}^{3}\cdot {\left\{{\text{PO}}_{4}^{3-}\right\}}^{2}.$$ (5)

Here, DCPD is dicalcium phosphate dihydrate, *β*TCP is beta-tricalcium phosphate, OCP is octacalcium phosphate, HAp is hydroxyapatite, and ACP is amorphous calcium phosphate.

In this report the {Ca^2+^} and {H^+^} were measured directly by ion-selective electrodes, leaving only estimates of {OH^−^} ({OH^−^} = *K*_w_/10^−pH^, where *K*_w_ is the dissociation constant for water), $\left\{{\text{PO}}_{4}^{3-}\right\}$, ${\text{\{HPO}}_{4}^{2-}\}$ and ionic strengths needed to calculate the *IAP*s. These activities were calculated, as described below, from the binding constants of the various inorganic phosphate binding moieties found in plaque fluid (Table 1) and the appropriate activity coefficients *γ*. The activity coefficients were calculated at an assumed ionic strength of 150 mmol/L for plaque fluid [14] using the Davies’ modification of the Extended Debye-Hückel Equation [15] and the Debye factor *A* (Table 1). Because the temperatures of the samples were not maintained during the measurements, the average room temperature of 22 °C was used in this calculation.

To calculate the $\left\{{\text{PO}}_{4}^{3-}\right\}$ and $\left\{{\text{HPO}}_{4}^{2-}\right\}$ from the total concentration of [PO_4_]_Tot_, the following mass balance equation was used:

$$\begin{array}{c} {\left[{\text{PO}}_{4}\right]}_{\text{Tot}}=\left[H_{3}{\text{PO}}_{4}\right]+\left[H_{2}{\text{PO}}_{4}^{-}\right]+\left[{\text{HPO}}_{4}^{2-}\right]+\left[{\text{PO}}_{4}^{3-}\right]\\ +\left[{\text{CaH}}_{2}{\text{PO}}_{4}^{+}\right]+\left[{\text{CaHPO}}_{4}^{0}\right]+\left[{\text{CaPO}}_{4}^{-}\right]. \end{array}$$ (6)

Utilizing the equilibrium expressions corresponding to the appropriate constants of Table 1, Eq. (6) can be rewritten as

$${\left[{\text{PO}}_{4}\right]}_{\text{Tot}}=\left\{{\text{HPO}}_{4}^{2-}\right\}\cdot \left[F\left(H,\gamma ,K\right)+\left\{{\text{Ca}}^{2+}\right\}\cdot G\left(H,\gamma ,K\right)\right],$$ (7)

where

$$F\left(H,\gamma ,K\right)=\frac{\left\{H^{+}\right\}}{K_{P_{1}}\cdot K_{P_{2}}}+\frac{\left\{H^{+}\right\}}{K_{P_{2}}\cdot \gamma_{H_{2}{\text{PO}}_{4}^{-}}}+\frac{1}{\gamma_{{\text{HPO}}_{4}^{2-}}}+\frac{K_{P_{3}}}{\left\{H^{+}\right\}\cdot \gamma_{{\text{PO}}_{4}^{3-}}},$$ (8)

and

$$G\left(H,\gamma ,K\right)=\frac{\left\{H^{+}\right\}\cdot K_{{\text{CaH}}_{2}{\text{PO}}_{4}^{+}}}{\gamma_{{\text{CaH}}_{2}{\text{PO}}_{4}^{+}}\cdot K_{P_{2}}}+K_{{\text{CaHPO}}_{4}^{0}}+\frac{K_{P_{3}}\cdot K_{{\text{CaPO}}_{4}^{-}}}{\left\{H^{+}\right\}\cdot \gamma_{{\text{CaPO}}_{4}^{-}}}.$$ (9)

Here the terms $K_{P_{1}}$, $K_{P_{2}}$, and $K_{P_{3}}$ in Eqs. (7)–(9) are the dissociation constants for phosphoric acid and the terms $K_{{\text{CaH}}_{2}{\text{PO}}_{4}^{+}}$, $K_{{\text{CaHPO}}_{4}^{0}}$ and $K_{{\text{CaPO}}_{4}^{-}}$ in Eq. (9) are the association constants for the indicated ion pairs and are presented in Table 1. The functions *F*(H, *γ*, *K*), *G*(H, *γ*, *K*) in Eq. (6) represent the phosphate binding by H^+^ and Ca^2+^, respectively. This equation can be expanded [*H*(H, *γ*, *K*), *I*(H, *γ*, *K*), …] to include other phosphate-binding substances such as sodium and magnesium, which were not measured for the current paper. The concentration and/or the binding constants of these other phosphate-binding substances appear to be small in comparison to the concentration of total phosphate in PF [19] and do not affect the computation significantly.

The quantity $\left\{{\text{HPO}}_{4}^{2-}\right\}$ is obtained by dividing both sides of Eq. (7) by the expression in the square brackets and from this result the activity of ${\text{PO}}_{4}^{3-}$ is calculated:

$$\left\{{\text{PO}}_{4}^{3-}\right\}=\frac{K_{P_{3}}\cdot \left\{{\text{HPO}}_{4}^{2-}\right\}}{\left\{H^{+}\right\}}.$$ (10)

The *IAP* for the Ca-PO_4_ minerals can then be calculated by use of Eqs. (1)–(5).

### 2.8 Simplified Calculation of the PF-*IAP*s for the Calcium-Phosphate Salts Suitable for Spreadsheets

Two observations about the phosphate ion activities in PF can lead to an acceptable estimate of the $\left\{{\text{PO}}_{4}^{3-}\right\}$ that greatly simplifies the calculation of PF-*IAP*s. Firstly, over the pH range likely to be found in PF, the concentrations of H_3_PO_4_ and ${\text{PO}}_{4}^{3-}$ species are very small and thus the concentrations of $H_{2}{\text{PO}}_{4}^{-}$ and ${\text{HPO}}_{4}^{2-}$ species can account for virtually all the phosphate in solution. Thus, in Eq. (8) the first and fourth terms can be taken as 0. Secondly, only a very small proportion of the total PO_4_ in PF is bound to calcium because of the relatively large concentration of phosphate at ≈ 10 mmol/L versus the much smaller concentration of calcium at ≈ 3 mmol/L. Sample calculations show that the $\left\{{\text{PO}}_{4}^{3-}\right\}$ is reduced by less than 10 % when the calcium is present at a concentration of 3 mmol/L and the pH of the PF is 7.5, therefore *G*(H, *γ*, *K*) = 0 (i.e., the value of Eq. (9) is 0.

Thus Eq. (7) becomes

$${\left[{\text{PO}}_{4}\right]}_{\text{Tot}}=\left\{{\text{HPO}}_{4}^{2-}\right\}\cdot F^{\prime }\left[H,\gamma ,K\right],$$ (11)

where

$$F^{\prime }[H,\gamma ,K]=\frac{\left\{H^{+}\right\}}{K_{P_{2}}\cdot \gamma_{H_{2}{\text{PO}}_{4}^{-}}}+\frac{1}{\gamma_{{\text{HPO}}_{4}^{2-}}}.$$ (12)

The quantity $\left\{{\text{HPO}}_{4}^{2-}\right\}$ is obtained by dividing both sides of Eq. (11) by the function *F'*[H, *γ*, *K*] and from this result the activity of ${\text{PO}}_{4}^{3-}$ is calculated from Eq. (10).

Spreadsheet representations that can be used to calculate $\left\{{\text{HPO}}_{4}^{2-}\right\}$ and $\left\{{\text{PO}}_{4}^{3-}\right\}$ at 25°C from the sample pH and the concentration of [PO_4_]_Tot_ at an ionic strength of 150 (mmol/L) are given in Eqs. (13) and (14). The numerical constants in these equations are the product of $K_{P_{2}}$ and $\gamma_{H_{2}{\text{PO}}_{4}^{-}}$ [6.46 × 10^−8^ $\left(K_{P_{2}}\right)$ multiplied by 0.7589 $\left(\gamma_{H_{2}{\text{PO}}_{4}^{-}}\right)=4.90\times 10^{-8}$]; $1/\gamma_{H_{2}{\text{PO}}_{4}^{2-}}$ [1/0.3318 = 3.0139]; and $K_{P_{3}}=4.47\times 10^{-13}$.

{HPO42−}=[PO4]Tot/[(10^−pH)/(4.90×10−8)+3.0139] (13)

{PO43−}=(4.47×10−13⋅{HPO42−})/(10^−pH). (14)

## 3. Results

### 3.1 Calcium Ionophore Evaluation

The [Ca^2+^] in whole pooled saliva determined by inverted macro Ca-ISE, protected with a dialysis membrane, was (0.88 ± 0.12) mmol/L (mean ± 1 standard deviation of the mean, *n* = 20). The [Ca^2+^] in saliva determined with micro Ca-ISEs that utilized neutral and charged carriers was (0.84 ± 0.17) mmol/L (*n* = 41) and (0.66 ± 0.20) mmol/L (*n* = 99), respectively. The paired difference in the [Ca^2+^] determined by the two types of micro Ca-ISEs of (0.24 ± 0.18) mmol/L (*n* = 41 pairs) was significant (*a* < 0.001). No significant difference was found in the [Ca^2+^] determined by the charged carrier type inverted macro calcium electrode and the microelectrodes with the neutral carrier (*a* > 0.1, *n* = 20 pairs). Because of the observed discrepancy in the values derived from the charged carrier micro ISEs versus those from the macro and neutral carrier ISEs, the only [Ca^2+^] results reported in this study are from analysis made with micro Ca-ISEs that contained the neutral carrier micro- or inverted macro Ca-ISEs.

### 3.2 Calcium Analysis of Plaque and Plaque Fluid

Table 2 shows the average [Ca^2+^] and [Ca]_Tot_ concentrations for PF determined in this study. For comparison the results of several previous studies are given. The pH, [PO_4_]_Tot_ concentration, and −log(*IAP*_HAp_) are also given in Table 2. Table 3 presents a comparison between the microelectrode determined values for pH and [Ca^2+^] for whole plaque and the PF recovered by centrifugation from the same whole plaque sample. No significant differences in the pH or [Ca^2+^] were caused by the centrifugation and separation of PF from the plaque mass (*a* > 0.1, Student *t*-test).

### 3.3 Comparison of the Values for $\left\{{\text{HPO}}_{4}^{2-}\right\}$ and $\left\{{\text{PO}}_{4}^{3-}\right\}$ in PF Calculated by the Rigorous and Simplified Methods

Table 4 gives the values for $\left\{{\text{HPO}}_{4}^{2-}\right\}$ and $\left\{{\text{PO}}_{4}^{3-}\right\}$ for a range of pH values from 5.0 to 7.5 when calculated by the rigorous and simplified methods as described in Eqs. (6)–(10) and Eqs. (11)–(14), respectively. For these calculations a constant ionic strength of 150 mmol/L and a constant [PO_4_]_Tot_ of 10 mmol/L for PF were assumed. For the rigorous calculation a constant {Ca^2+^} of 1.0 mmol/L was assumed. The simplified method consistently yielded an overestimate of the activity values for phosphate species. The percent difference in the activities of these species is related to the pH with the smallest difference at lower pH and greater as the pH increases. The calculated difference ranged from −1.1 % to −20.6 % over the pH range 5.0 to 7.5. At pH 7.5 the difference in the concentration calculated for $\left\{{\text{PO}}_{4}^{3-}\right\}$ resulted in a change in the calculated −log(*IAP*_HAp_) of 0.244 [44.203 (rigorous) −43.959 simplified) = 0.244], while at pH 5.0 the difference in the concentration calculated for $\left\{{\text{PO}}_{4}^{3-}\right\}$ resulted in a change in the calculated −log(*IAP*_HAp_) of 0.014 [59.279 (rigorous) −59.265 (simplified) = 0.014].

## 4. Discussion

The evaluation of Ca-ISE ion exchangers found significant differences in the responses between the neutral carrier and charged carrier types of microelectrodes when determining the activity of calcium in whole pooled saliva. These differences in response were not always consistent, resulting in a wider range of responses between electrodes containing the charged carrier. One interesting observation was that microelectrodes of the charged carrier type that were constructed with very fine tips (tip opening ≤1 μm) responded similarly to the micro ISEs made with the neutral-carrier. These very fine-tipped ISEs tended to have a shorter useable lifetime and required more time to stabilize between readings. Macro electrodes that contained the charged carrier that were protected with dialysis membranes always gave the same response as the neutral carrier type microelectrodes. It is not clear what caused the difference in the responses between the neutral carrier and charged carrier type of ISE that was not protected. However, because the effect could be removed by protection of the ion exchange fluid with a dialysis membrane, it is probable that the charged-carrier suffered some interference from macromolecules. It is suggested that to assure reliable results, any calcium electrode be thoroughly checked in saliva samples of known calcium activity, or against a macro Ca-ISE that has been protected against macromolecules, before measurement in plaque or plaque fluid samples.

No significant differences were found between the pH and [Ca^2+^] measured with microelectrodes in the extracellular fluid of whole plaque (before centrifugation) and the fluid obtained by centrifugation of those same samples (Table 3). This result agrees with earlier results where the potassium activity was determined before and after centrifugation [20]. This indicates that the composition of plaque fluid derived from fresh whole plaque by centrifugation is not significantly altered during the process of centrifugation. The composition results for pH, {Ca^2+^}, [Ca]_Tot_, and [PO_4_]_Tot_ for starved (no food for more than 12 h) PF compare favorably with earlier reports [6, 7] (see Table 2).

The calculation of the ion activity product with respect to Ca-PO_4_ minerals can be greatly simplified when the activities of the mineral ions are known, {OH^−^}, {Ca^2+^}, $\left\{{\text{HPO}}_{4}^{2-}\right\}$, and $\left\{{\text{PO}}_{4}^{3-}\right\}$. The activities of OH^−^ and Ca^2+^ were determined directly via ion-selective electrode and the [PO_4_]_Tot_ was determined spectrophoto-metrically. The activities of the phosphate species $\left\{H_{2}{\text{PO}}_{4}^{-}\right\}$, $\left\{{\text{HPO}}_{4}^{2-}\right\}$, and $\left\{{\text{PO}}_{4}^{3-}\right\}$ were estimated from the [PO_4_]_Tot_ with good accuracy even when the effect of binding by cations to phosphate was ignored (Table 4). The benefit of such simplification is that the activity of the phosphate species, and thus the *IAP* with respect to the calcium-phosphate salts, can be calculated with good accuracy directly on a spreadsheet by use of simple equations such as Eqs. (13) and (14). The difference introduced from such assumptions is about 20 % in the activity of a phosphate species at pH 7.5 but much less at lower pH values. Even at the high pH where this systematic difference is largest, the difference in the calculation of the −log(*IAP*_HAp_) (0.244) is smaller than the uncertainty calculated from the uncertainties inherent in the analytical techniques for pH, [PO_4_]_Tot_, and {Ca^2+^}. Propagation of uncertainty (sometimes called propagation of error) calculations based on the uncertainties in the determination of pH, [PO_4_]_Tot_ Tot, and {Ca^2+^} yields an estimate of ±0.3 [16] for the smallest uncertainty that can be determined for −log(*IAP*_HAp_) with the analytical methods used for this study. The reason that the systematic difference is small is that the abundance of phosphate in PF is much higher than that of calcium (Table 2), and therefore the calcium binding cannot reduce the phosphate activity by a large amount. The use of an ion selective electrode that measures the activity of ${\text{HPO}}_{4}^{2-}$ [21] in solution will further simplify and improve the uncertainty of *IAP* estimates with respect to the Ca-PO_4_ salts.

## Figures and Tables

**Table 1:** Dissociation, association, and Debye constants at 22 °C

Dissocciation constant *K*	Reaction	*K*	Reference
$K_{p_{1}}$	$H_{3}{\text{PO}}_{4}⇄H^{+}+H_{2}{\text{PO}}_{4}^{-}$	7.33 × 10^−3^	[22]
$K_{P_{2}}$	$H_{2}{\text{PO}}_{4}^{-}⇄H^{+}+{\text{HPO}}_{4}^{2-}$	6.20 × 10^−8^	[22]
$K_{P_{3}}$	${\text{HPO}}_{4}^{2-}⇄H^{+}+{\text{PO}}_{4}^{3-}$	4.07 × 10^−13^	[22]
*K*_w_	$H_{2}O⇄H^{+}+{\text{OH}}^{-}$	8.02 × 10^−15^	[23]
Debye (*A*)		0.5088	[24]

Association constants *K*_a_	Ion pairs	*K*_a_	Reference

$K_{{\text{CaH}}_{2}{\text{PO}}_{4}^{+}}$	${\text{Ca}}^{2+}+H_{2}{\text{PO}}_{4}^{-}⇄{\text{CaH}}_{2}{\text{PO}}_{4}^{+}$	8.59	[22]
$K_{{\text{CaHPO}}_{4}^{0}}$	${\text{Ca}}^{2+}+{\text{HPO}}_{4}^{2}⇄{\text{CaHPO}}_{4}^{0}$	258.0	[22]
$K_{{\text{CaPO}}_{4}^{-}}$	${\text{Ca}}^{2+}+{\text{PO}}_{4}^{3-}⇄{\text{CaPO}}_{4}^{-}$	2.9 × 10^7^	[25]

**Table 2:** The mean and standard deviation of the mean for pH, [Ca^2+^], [Ca]_Tot_ and [PO_4_]_Tot_ of plaque fluid

	This study	Ref. [6]	Ref. [7]
pH^b^	6.98 ± 0.28 (*n* = 15)	6.78 ± 0.41 (*n* = 63)	7.02 ± 0.05 (*n* = 13)
[Ca^2+^] (mmol/L)	0.74 ± 0.19 (*n* = 15)	0.85 ± 0.52 (*n* = 63)	1.873^c^
[Ca]_Tot_ (mmol/L)	3.9 ± 2.0 (*n* = 15)	3.1 ± 1.7 (*n* = 55)	2.8 ± 0.2 (*n* = 13)
[PO_4_]_Tot_ (mmol/L)	10.2 ± 5.4 (*n* = 15)	11.5 ± 3.0 (*n* = 63)	13.9 ± 1.9 (*n* = 13)
−log(*IAP*_HAp_)^d^	49.536	50.164	46.953

a Data from caries-free group.

b Average pH is calculated from (ΣpH)/*n*.

c Calculated from the ion composition and only considers calcium binding by phosphate and organic acid anions.

d Calculated from the average values for pH, {Ca^2+^}, and [PO_4_]_Tot_ and assuming an ionic strength of 150 (mmol/L) for PF. The −log(*IAP*_HAp_) at saturation is 58.554 [26] thus PF is highly supersaturated with respect to this mineral.

**Table 3:** The mean and standard deviation of the mean for pH and [Ca^2+^] concentrations in whole plaque samples and plaque fluid derived by centrifugation

	Whole plaque	Plaque fluid^a^
pH	6.71 ± 0.50 (*n* = 24)	6.88 ± 0.33 (*n* = 6)
[Ca^2+^] (mmol/L)	1.3 ± 1.0 (*n* = 20)	1.2 ± 0.3 (*n* = 5)

a No significant differences between whole plaque and plaque fluid values for pH or [Ca^2+^] (*a* ≥ 0.1, Student *t*-test).

**Table 4:** Comparison of $\left\{{\text{HPO}}_{4}^{2-}\right\}$, and $\left\{{\text{PO}}_{4}^{3-}\right\}$ in PF calculated by the rigorous and simplified methods over a range of pH values with ionic strength of 150 (mmol/L) at 22°C

pH	Simplified method^a^		Rigorous method^a^,^b^		% difference ${\left\{{\text{HPO}}_{4}^{2-}\right\}}^{c}$	% difference ${\left\{{\text{PO}}_{4}^{3-}\right\}}^{d}$
	$\left\{{\text{HPO}}_{4}^{2-}\right\}$ (mol/L)	$\left\{{\text{PO}}_{4}^{3-}\right\}$ (mol/L)	$\left\{{\text{HPO}}_{4}^{2-}\right\}$ (mol/L)	$\left\{{\text{PO}}_{4}^{3-}\right\}$ (mol/L)		
5.0	4.647 × 10^−5^	1.893 × 10^−12^	4.598 × 10^−5^	1.873 × 10^−12^	−1.1	−1.1
5.5	1.426 × 10^−4^	1.873 × 10^−11^	1.409 × 10^−4^	1.815 × 10^−11^	−1.2	−3.2
6.0	4.129 × 10^−4^	1.682 × 10^−10^	4.053 × 10^−4^	1.651 × 10^−10^	−1.9	−1.9
6.5	1.030 × 10^−3^	1.327 × 10^−9^	9.928 × 10^−4^	1.279 × 10^−9^	−3.7	−3.8
7.0	1.954 × 10^−3^	7.957 × 10^−9^	1.802 × 10^−3^	7.338 × 10^−9^	−8.4	−8.4
7.5	2.727 × 10^−3^	3.512 × 10^−8^	2.261 × 10^−3^	2.912 × 10^−8^	−20.6	−20.6

a Calculated with [PO_4_]_Tot_ = 10 (mmol/L).

b Calculated with {Ca^2+^} = 1.0 (mmol/L).

c % difference calculated as $\Delta \{{\text{HPO}}_{4}^{2-}\}=100.\frac{\left({\left\{{\text{HPO}}_{4}^{2-}\right\}}_{\text{Rigorous}}-{\left\{{\text{HPO}}_{4}^{2-}\right\}}_{\text{Simplified}}\right)}{{\left\{{\text{HPO}}_{4}^{2-}\right\}}_{\text{Rigorous}}}$.

d % difference calculated as $\Delta \{{\text{PO}}_{4}^{3-}\}=100.\frac{\left({\left\{{\text{PO}}_{4}^{3-}\right\}}_{\text{Rigorous}}-{\left\{{\text{PO}}_{4}^{3-}\right\}}_{\text{Simplified}}\right)}{{\left\{{\text{PO}}_{4}^{3-}\right\}}_{\text{Rigorous}}}$.

## Footnotes

1 Certain commercial equipment, instruments, or materials are identified in this paper to foster understanding. Such identification does not imply recommendation or endorsement by the National Institute of Standards and Technology, or the American Dental Association Health Foundation, nor does it imply that the materials or equipment identified are necessarily the best available for the purpose.
